# From prospective clinical trial to reducing social inequalities in health: The DESSEIN trial, concept and design of a multidisciplinary study in precarious patients with breast cancer

**DOI:** 10.1186/s12889-019-7611-6

**Published:** 2019-11-04

**Authors:** Charlotte Ngô, Aurélia Dinut, Audrey Bochaton, Hélène Charreire, Caroline Desprès, Sandrine Baffert, Fabrice Lécuru, Gilles Chatellier

**Affiliations:** 1 Centre Expert Oncologie Gynécologique et Sénologie, Hôpital Européen Georges Pompidou, AP-HP, Paris, France; 2 Faculté de Médecine, Université Paris-Descartes, Sorbonne Paris Cité, Paris, France; 3 Clinical Research Unit and CIC 1418 INSERM Hôpital Européen Georges Pompidou, AP-HP, Paris, France; 4 Health Geography, Université Paris Nanterre (UPN), UMR7533 Ladyss, Nanterre, France; 5 Université Paris-Est, Lab’Urba, UPEC, Créteil, France; 6 Health Economist, Department of Health Economy, Cemka, Paris, France; 7 Epidemiologist, Clinical Research Unit, and CIC 1418 INSERM Hôpital Européen Georges Pompidou, AP-HP, Paris, France

**Keywords:** Social inequalities in health, Breast cancer, Precariousness, Socio-economic deprivation, Geographic information system, Health economist, Health geographer, Epidemiologist, Anthropologist, Qualitative interviews, Stage at diagnosis

## Abstract

**Background:**

In France during the last 15 years, precariousness among women has increased. In breast cancer, precariousness has been associated with an increase in mortality, but the links between precariousness, stage at diagnosis and care pathway are little explored. Our study aims to evaluate the impact of precariousness on care pathways, treatment and recovery phase according to a multidisciplinary analysis.

**Methods and design:**

Comparative prospective observational multicenter study of exposed / unexposed category. Patients with breast cancer are recruited in the Ile de France area. Three scores are used to identify precarious patients. Precarious patients are matched to non-precarious patients by age group. Questionnaires are distributed to patients at different times of care. The main objective is to compare the stage of the disease at diagnosis between two groups. The secondary objectives are: comparison of socio-economic and geographical characteristics, direct and indirect costs, personal trajectories of care and health. Analysis include multidisciplinary approaches. A geographical information systems method will evaluate the accessibility to health facilities and the characteristics of the places of residence of the patients. An anthropological analysis will be conducted through observation of consultations and semi-directed interviews with patients. These methods will allow to analyze the diagnostic and therapeutic routes, placing it in a life history and an economic, socio-cultural and health environment. The economic analysis will include a comparison of direct, indirect costs and out-off pocket costs, from the patient’s point of view and from the societal perspective.

**Discussion:**

Conducted in a clinical setting and coupled with a qualitative study, this study will provide a better understanding of how contextual factors, combined with individual factors, can influence the course of health and thus the stage of the disease at diagnosis. The multidisciplinary approach, involving clinicians, geographers, an anthropologist, an economist and a health epidemiologist, will allow a multidimensional approach to the impact of precariousness on breast cancer.

**Trial registration:**

ClinicalTrials.gov Identifier: NCT02948478 registered October 28, 2016. ID RCB: 2016-A00589–42. protocol version: 2.1. decembre 13, 2018.

## Background

Breast cancer is the most frequent cancer among women with around 50,000 new cases per year in France. There is a social gradient in incidence and mortality with a lower life expectancy of persons belonging to the most disadvantaged socio-economic groups.

Socioeconomic and geographical inequalities in cancer mortality have been widely described in European Countries and in the US [1–3]. Until the 70’s, breast cancer was characterized by its higher incidence among women with high educational level of than in women with a low-level educational level. Inequalities in mortality were therefore difficult to assess, since there was a combination of different cancer incidences favoring women with a low socioeconomic status and survival difference favoring women with a high socioeconomic status. In France, during the 2000s, the situation regarding health inequalities among women has worsened [4]. Presently, the increased incidence in women with a high-educational level tends to disappear, which strengthen the drawback of the most disadvantaged women [5, 6]. Prognosis is strongly related to stage at diagnosis, with a 5-year overall survival rate decreasing from 98% for stage 1 to 20% for stage 4 [7]. It has recently been shown in UK that breast cancer is more likely to be diagnosed in advanced stage among precarious patients, resulting in decreased survival in those patients. These inequalities are widening when breast cancer leads to significant decreases in income, additional costs and difficulties to re-entering the work world [8].

Otherwise, little is known about the relationship between precariousness, breast cancer stage at diagnosis and breast cancer treatment course. Precariousness is a multifactorial concept. The definition of precariousness elaborated by J. Wresinski and adopted by the French High Committee for Public Health is “the lack of one or more of the securities, especially that of employment, allowing individuals and families to assume their professional, family and social obligations and to enjoy of their fundamental rights “[9]. Precariousness occurs when the socio-economic, housing, financial reserves, cultural, educational and professional qualifications, means of associative and political participation are unfavorable, leading to variable forms of vulnerability. Precariousness is therefore rather a progressive and potentially reversible process than a social category [10]. Our aim is therefore to analyze not only the effects of precariousness on health indicators related to breast cancer but also, reciprocally, the effects of the disease as a factor of both social and economic vulnerability.

In the light of recent studies, several elements occurring at different times of the care pathway can help understanding precariousness impact.

First, precarious women have a poor access to screening (mammography) which is one of the causes of a diagnosis delay. Thus, in France, it has been shown that the women’s economical and geographical situation is a significant determinant of breast cancer screening and that diverse situations of insecurity could lead to a renunciation to breast cancer screening [11]. Even in the case of clinical symptoms (palpable tumor, for example), some women may delay the medical consultation. Inequalities in access to screening need to be further documented, to understand if this is a consequence of a lower demand from disadvantaged social groups, or a consequence of an inability of those groups to access to the health care services, or both.

Second, there may be a delay between confirmed diagnosis and initiation of treatment varying with the medical diagnosis context (opportunistic screening, organized screening, clinical symptoms), varying availability, accessibility and coordination of healthcare resources in geographical areas, and finally, individual factors.

Third, variations in access and quality of care for people with breast cancer could also explain differences in survival rates. The French health insurance system ensures patient access to the most effective treatments, regardless of income level and type of insurance [12]. This provides rather good healthcare access compared to other western countries, but also hide greater inequalities [4]. It has also been shown an association between socioeconomic status and quality of oncology care, with, for example, fewer participation of low-income patients in clinical trials suggesting unequal access to the newest cancer treatments [13]. The remoteness of specialized centers and living in an area with significant economic and social precariousness can reduce significantly the likelihood of patients to access to the best quality of care. From an economic point of view, precariousness increases both hospital length of stay and cost [14]. Thus, in breast cancer, Medicaid patients who underwent surgical resection (+ − reconstruction) had a higher length of stay than private insured patients [15].

The description and analysis of the care pathway has been the subject of numerous works including sociological and anthropological studies, which invariably note its diversity, plurality and complexity. This complexity is part of our multicultural contemporary societies [16]. The context of free movement of ideas, people and products causes a multiplication of health actors from various backgrounds. Anthropological studies about care pathway in precarious situations are less common [17, 18]. Moreover, most of epidemiologic and health economics studies are focused on the economic determinants possibly linked to the different forms of social protection. However, as we mentioned earlier, precariousness is not only connected to the material conditions of existence. Precariousness also refers to relationships that patients have with their lifetime, with others, with their own body, relationships that determine their behaviors. In addition, the social and health environment can facilitate or hinder the access to specialized services and professionals. Reasons of renunciation or delay in screening and care have to be described in the specific setting of the course of breast cancerand of breast cancer treatment [17].

There are few studies in France about socioeconomic and geographic inequalities in cancer and most of the studies are on data registries with incomplete clinical and demographical data [19]. In addition, there is a consequent number of studies about cancer in social or anthropological perspectives, but very few have taken an interest to precariousness [20].

Our study is a specifically designed study in the field of social and human sciences grounded on a multidisciplinary approach and conducted in a clinical environment allowing avoiding the weaknesses of studies performed on registers. Our study is designed to assess the impact of precariousness on the history of breast cancer, on the treatment and the rehabilitation phases in a multidisciplinary contextual analysis. Geographical, social, economic and anthropological analysis will be conducted at different times of the care pathway. The final aims are: first to implement corrective measures for an appropriate breast cancer diagnosis in precarious women to design care management adapted to each situation of insecurity, second, to warn policy makers on these precarious situations and the impact of possible correctives measures.

## Methods and design

### Study design and outcomes

#### Study design

The DESSEIN (“**D**isparités **E**conomiques et **S**ociales et cancer du **SEIN”**) is an academic, investigator-driven, prospective observational multicenter cohort study comparing patients “Exposed” to precariousness to patients “unexposed” to precariousness.

Three scores of precariousness will be applied to patients for determining precariousness:

- EPICES score [21]
- Pascal score [22]
- The most relevant basic needs retained in the study of the French version of EDI (European Deprivation Index) [23].

Precariousness will be defined by identification of this condition by at least one of the three scores. Absence of precariousness is defined by absence of identification of this condition by the 3 scores.

Each precarious patient will be matched to a non- precarious patient belonging to the same age group, regardless of the center. Each woman will receive breast cancer treatment according to stage of the disease and to local multidisciplinary procedures of each center. In France, treatment decisions are made during multidisciplinary meetings in agreement with the national and international recommendations and/or with the local referential.

Deprived patients are compared to non-deprived patients in the breast cancer care pathway.

#### Outcomes

The primary outcome is the comparison of the stage of disease at diagnosis (according to the TNM classification) between precarious and non-precarious patients.

Secondary outcomes are:

- to describe the socio-economic situations and geographical context between precarious and non-precarious patients in quantitative and qualitative approaches,
- to describe diagnostic and therapeutic pathways of women with breast cancer including the diagnostic phase, the treatment phase and the phase of post-treatment and rehabilitation,
- to compare those pathways between precarious and non-precarious women,
- to compare in both groups of women the direct and indirect costs of care and the out-of-pocket costs.

### Ethical and regulatory aspects

The study is conducted in accordance with the guidelines of the Declaration of Helsinki. This protocol is part of a research to evaluate routine care as defined by art No. 2004–806 of 9^th^August 2004 on public health policy and its implementing decree (No. 2006–477) of 26th April 2006. (Mandate: Articles L.1121–1, section 2 and R1121–3 of the French Public Health Code). In each center participating, patients will be asked by their physician to sign an informed consent. The study was approved by the Ethics Committee-Paris Ile de France III on 24 May 2016.

### Funding

The study was supported by a grant (2015–1-PL SHS-01) of INCA (French national cancer institute).

### Patient eligibility and data collection

All patients seeking treatment for breast cancer, regardless of the cancer stage, are invited to participate in this study.

Women with a breast cancer over 18 years of age are eligible for the study, if they have no history of cancer (any site) treated in the previous five years and no associated cancer at time of diagnosis. Inclusion and exclusion criteria are summarized in the Table 1.

**Table 1:** Inclusion and exclusion criteria

Inclusion criteria	Exclusion criteria
Patient over 18	Patient under 18
Breast cancer histologically proven	Patient under guardianship
Written informed consent	Patient treated for a cancer in the 5 previous years
	Patient with treated at the same time for another cancer

Women will be recruited in breast cancer centers of the Ile de France region. Centers participating to the study are listed in the Table 2. These include University hospitals, public hospitals, private non-for-profit hospitals and private for-profit hospitals. These care centers are distributed in all districts of the Ile de France region to cover all geographical conditions.

**Table 2:** Centers participating in the DESSEIN study

Name of the center	City	Type of health care center
Hôpital Européen Georges Pompidou	Paris	Public
Hôpital Saint Louis	Paris	Public
Hôpital de Marne la Vallée	Marne-La-Vallée	Public
Hôpital André Mignot	Versailles	Public
Hôpital Delafontaine	Saint-Denis	Public
Clinique Hartmann	Neuilly	Private
Hôpital Les Peupliers	Paris	Private
Clinique St Faron	Mareuil-les-Meaux	Private
Centre hospitalier Intercommunal de Créteil	Créteil	Public
Centre hospitalier Intercommunal de Poissy-St Germain	Poissy	Public
Centre hospitalier sud francilien	Corbeil-Essonnes	Public
Hôpital Lariboisière	Paris	Public
Centre hospitalier Victor Dupouy	Argenteuil	Public
Hôpital Privé Paul D’Egine	Champigny sur Marne	Private
Hôpital Saint Joseph	Paris	Private
Institut de Cancérologie Paris Nord	Sarcelles	Private
Clinique Claude Bernard	Ermont	Private
Centre hospitalier Marie-Thérèse	Paris	Private
Hôpital Kremlin Bicêtre	Le Kremlin Bicêtre	Public
Centre hospitalier René Dubos	Pontoise	Public
Clinique de l’Estrée	Stains	Private

### Assessments

#### Geographical and anthropological analysis

The questionnaires analysis will be a socio-anthropological, socio-economic and geographical multidisciplinary analysis that will compare individual data with contextual data and clinico- pathological data, according of the levels of precariousness of the patients.

For geographical analysis, Health care and social environment will be assessed with Geographic Information Systems (GIS) based on available Geo-referenced databases (INSEE, IGN, FINESS) at neighbourhood level (IRIS) for the Ile-de-France region (n IRIS = 5261). Briefly, GIS can be defined as computer-based methods and tools which, via different information sources, enable organising, managing and combining spatial and thematic data, and representing and analysing results according to geographic location [24].

Anthropological analysis will be performed through observation of consultation and patients interviews. The observation of consultations will provide useful elements for the global analysis of care pathways (professional practices variability in behavior, including style of communication, information to women, understanding of the patient’s needs and choice of care, those elements related to patient’s communication). Different dimensions will be explored in individual interviews that can help understand the attitudes of women towards screening and their behavior in relation to the disease, the treatment, the professionals and the impact in everyday and professional life. Interviews will be conducted on a small number of patients in order to describe deeply the healthcare pathways and articulate different determinants of attitudes and behaviors with environment (medical, social, geographical) and context. An inductive approach in interviews is likely to provide news elements of understanding of the health care pathways. Interviews are performed by an anthropologist.

Study procedures are summarized in Table 3.

**Table 3:** Initial assessment and follow-up visits

Actions	Selection visit/ inclusion visit	Baseline assessment T0	T1 3 months	T2 6 months	T3 12 months End of research
Informed consent	X				
Inclusion fax	X				
History	X				
Clinical and radiological exam^a^		X			
Para-clinical exam^b^	X				
Questionnaires		X	X	X	X
Interview^c^		X	X	X	X

^a^clinical and radiological TNM

^b^histological proof of breast cancer

^c^One, two or three interviews, at any time between T0 and T3

#### Economic analysis

**Fig. 1:**
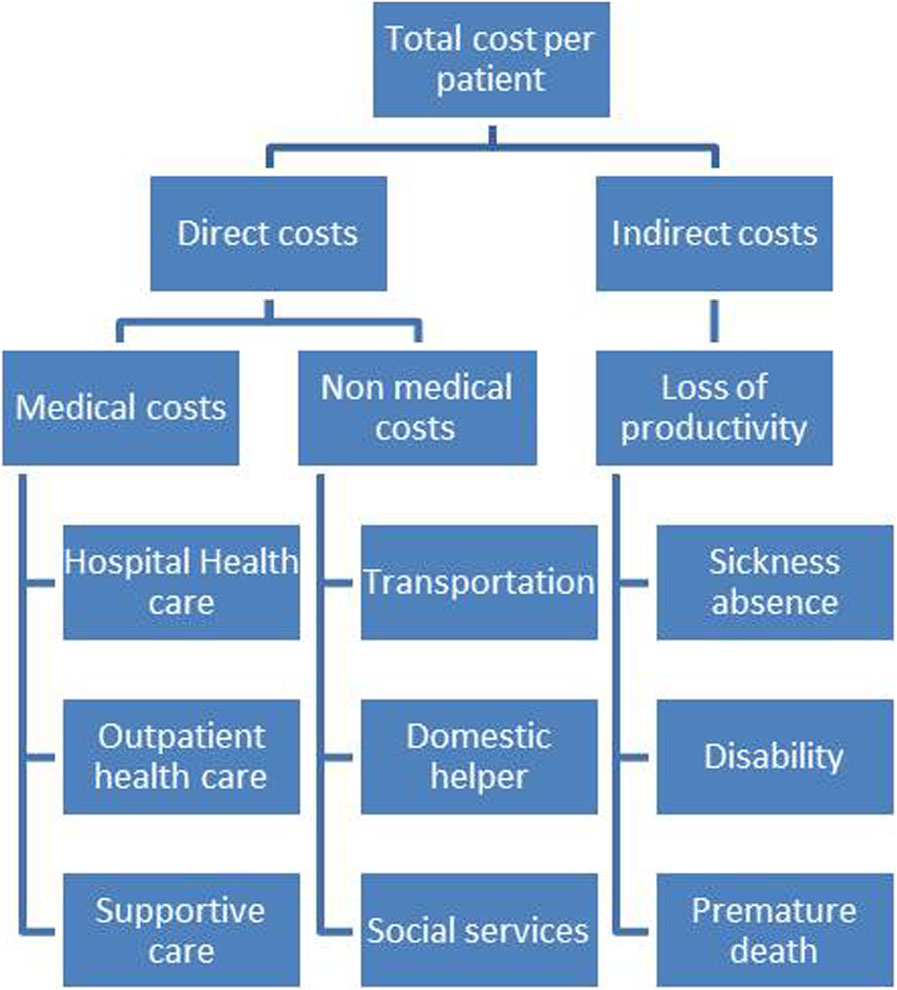
Types of costs. Total costs per patient include direct costs and indirect costs. Direct costs include medical and non-medical costs, indirect costs are the costs for the society, mainly the loss of productivity

The economic analysis will describe and compare the costs between the two groups of patients. The first part of analysis will reply to the question “is the precariousness in breast cancer induces over-costs from the society perspective?” This part will include the direct costs (medical direct costs (hospital and ambulatory costs) and nonmedical direct costs (transportation, home care services, social services)) and the indirect costs (productivity losses) [25, 26]. Productivity losses will be measured in terms of lost wages using human capital approach [27, 28]. The second part will reply to the question “Is the precariousness in BC induces differences in terms of out-of-pocket health expenses from patient’s perspective?” The out-of-pocket costs for treatments and follow-up, consultations with other practitioners, home help, clothing, and natural health products will be estimated, with information collected primarily from patient questionnaires before the surgery (T0) and at 3 and 6 months (T1, T2). Different types of costs are summarized in Fig. 1.

### Data management and statistical aspects

#### Data management

Patient data will be recorded using CleanWeb® electronic Case Report Form (eCRF) The following data will be retrieved from the patient’s medical record by trained personnel: demographic characteristics, diagnosis characteristics, imaging, stage of disease, treatments received (surgery, radiotherapy, chemotherapy, endocrine therapy), pathology, delay between diagnosis and first treatment.

Questionnaires will be distributed to all women to assess socio-economic and geographical determinants at inclusion and at 3, 6, and 12 months of follow-up.

- Inclusion: the questionnaire administered at baseline, before beginning treatment will comprise elements allowing calculation of the three precariousness scores allowing to identify women with and without precariousness.
- The questionnaires administered at 3, 6 and 12 months will focused on the health care renunciation, out-off pocket costs, return to work, rehabilitation. The last questionnaire at 12 months will also include the 3 scores of precariousness to determine if the disease worsened or not socio-economic deprivation.

#### Determination of sample size

Our hypothesis is that the proportion of stage 1 patients will be 50% among precarious patients versus 60% among non-precarious, a 10% absolute difference. To detect such a difference with a 90% power, 520 patients are needed (calculation made with NQUERY Advisor software). Therefore, our targeted sample size is 1040 patients (520 in each group). Inclusion period will last 36 months. Subject’s length of participation is 12 months. We recruited 20 participating sites in order to be able to include 1040 patients.

#### Statistical analysis

The strategy for design and analysis will be made in compliance with the CONSORT statement (http://www.consort-statement.org/).

Variables will be compared between the two groups according to standard tests: Student’s t test or nonparametric Wilcoxon test for quantitative parameters based on the variable distribution and chi-square test or Fisher test for proportions. The results will be presented as mean ± one standard deviation if the parameter follows a normal distribution and median [interquartile range] if the distribution is not normal for quantitative parameters. For qualitative parameters, the results will be presented as numbers (proportions).

The primary endpoint analysis will be carried out by a two-sided chi-square test on proportions. Odds ratio and its 95% confidence interval will be presented.

For secondary endpoints, the same principle will be applied for qualitative variables. For quantitative variables the mean difference and its 95% confidence interval will be presented.

All statistical analysis will be carried with SAS and R software, and the statistical significance level will be (two-sided) 0.05.

#### Quality control

A Clinical Research Associate (CRA) appointed by the sponsor will be responsible for the proper conduct of the research, for collecting and documenting, recording and reporting the data generated in writing, in accordance with the Standard Operating Procedures applied within the sponsor and in accordance with the French Good Clinical Practices as well as with the legislative and regulatory provisions in force. The sponsor’s CRAs will perform regular monitoring visits to the investigating centers at a rate corresponding to the patient follow-up schedule and enrolment rate. An audit can be carried out at any time by individuals appointed by the sponsor and not associated with the research directors. The objective of the audit is to ensure the quality of the research, the validity of the results and compliance with the legislation and regulations in force.

## Discussion

DESSEIN is one of the first prospective multidisciplinary study collecting real-life data on precariousness and breast cancer. Monitoring patients since the onset of the disease and up to 12 months will allow analyzing the whole pathway of care and rehabilitation based on individual data in all phases. Multi-dimensional and contextual analyses are among the strengths of our study. The multidisciplinary approach involving clinicians, geographers, anthropologists, health economists and epidemiologists, will allow an overview of the impact of precariousness on breast cancer management and the role of the different aspects of precariousness. The first patient was included on 15 December 2016. Twenty centers are now active and about 800 patients have been recruited up to June 2019.

### Expected results and perspectives

The expected results of this study are to assess the extent of difference in the initial stage of the disease between precarious and non-precarious women. In addition we will be able to describe the different types of precariousness and their impact on the course of the disease, during treatment and during rehabilitation. Finally we will be able to analyze the relationships between geographical, economic, social and anthropological vulnerability and clinico-pathological prognostic data in both groups of patients.

On a longer perspective, this study will allow to build new tools for a better diagnosis of precariousness in all its dimensions, to design corrective measures and to warn policy makers. These corrective measures could be: local measures (opening up isolated areas to compensate for low medical density), social measures (systematic referral of patients to social services of the hospital, work on perceptions of the disease and treatment), medical and economic measures (promoting participation in clinical trials, provide treatment of side effects, facilitate access to supportive care).

## Data Availability

Not applicable as the trial is ongoing, data are not yet available. No article presenting results of the study has been published. When available, the de-identified dataset will be transmitted to the researchers (all co-authors of this manuscript, and only co-authors of this manuscript) but not to all the investigators.

## Abbreviations

BC: Breast cancer
eCRF: e-case report form
EDI: European deprivation index
EPICES: Evaluation de la Précarité et des Inégalités de santé dans les Centres d’Examens de Santé
FINESS: Fichier National des Etablissements Sanitaires et Sociaux
GIS: Geographical information systems
IGN: Institut national de l’information géographique et forestière
INSEE: Institut national de la statistique et des études économiques
IRIS: Ilots Regroupés pour l’Information Statistique
